# Supplementary material for: The sources of atmospheric black carbon at a European gateway to the Arctic
Source: Nat Commun. 2016 Sep 15;7:12776. doi: 10.1038/ncomms12776 (PMC5027618; doi:10.1038/ncomms12776)
Supplement: Supplementary Information — Supplementary Figures 1-7, Supplementary Tables 1-9, Supplementary Notes 1 and Supplementary References [file ncomms12776-s1.pdf]

Supplementary Figures

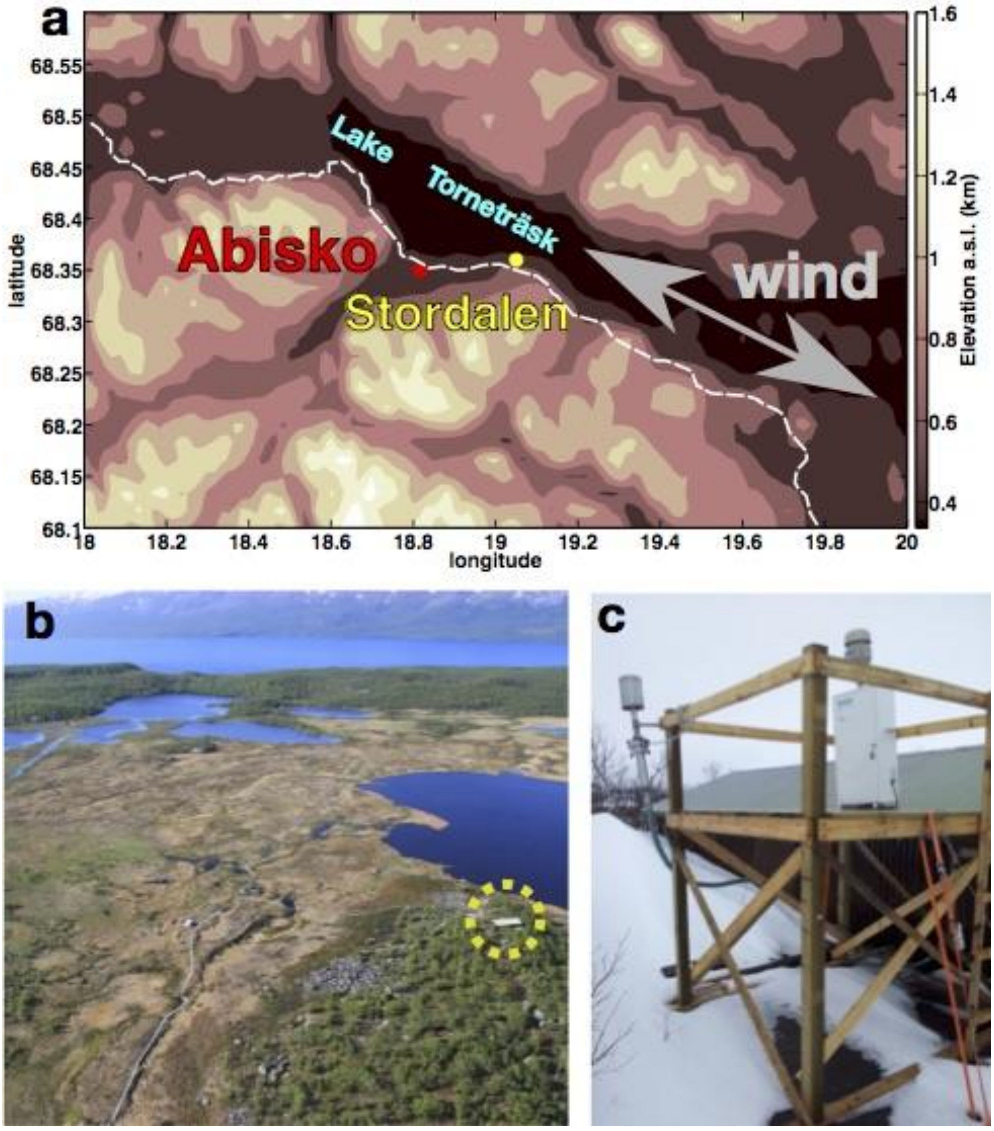

Supplementary Figure 1 Sampling site in detail. **a**, Elevation map (km scale) of the sampling site, Stordalen Mire (red); Abisko village (yellow), the road (dashed white line) and Lake Torneträsk. The arrow shows the main prevailing wind directions. **b**, Aerial view over Stordalen mire, looking northwards, with Lake Torneträsk in the background (photo by Niklas Rakos, Swedish Polar Research Secretariat). The sampling site, next to the cottage (Stordalen Villa), is marked with a yellow dashed circle. **c**, The two inlets of

the TSP (left) and $PM_{2.5}$ (right) samplers, mounted on top of a platform, overlooking the "Villa" and the mire.

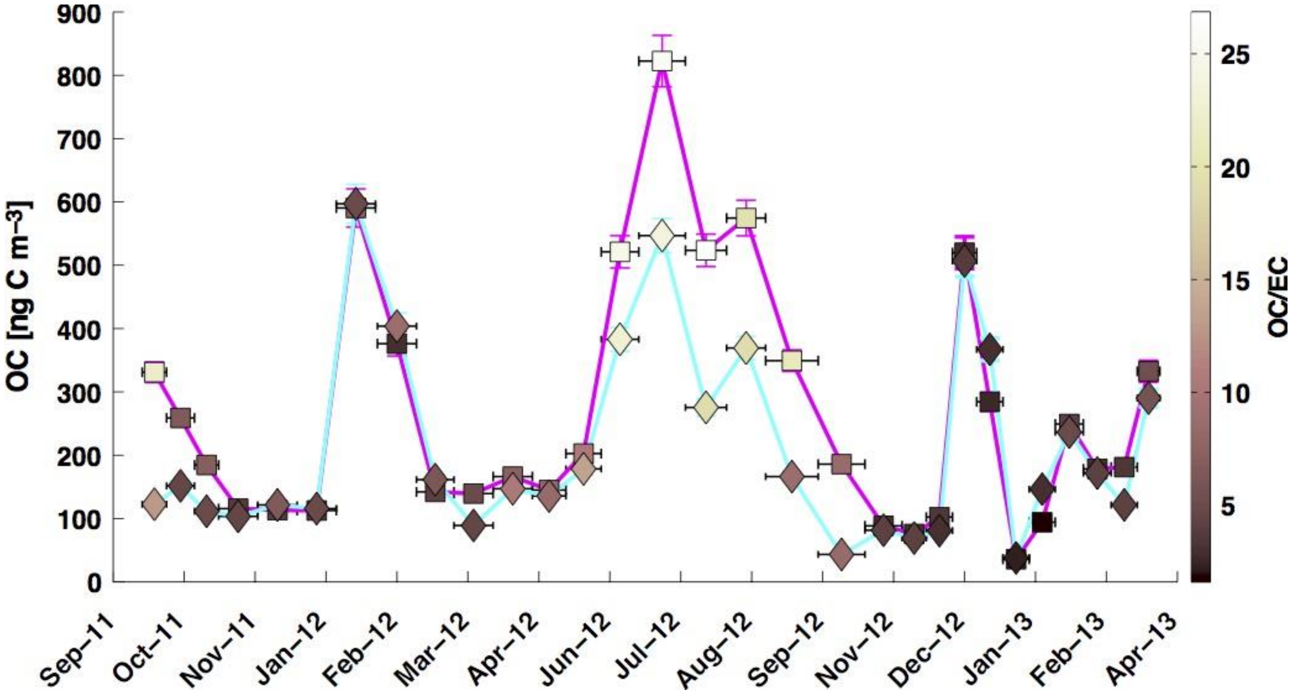

Supplementary Figure 2 Aerosol organic carbon concentration. For TSP (mangenta line and square symbols) and $PM_{2.5}$ (cyan line and diamond symbols). The colorbar represents the OC/EC fraction for each sample. Horizontal bars indicate duration of sampling. Vertical bars indicate standard deviations of the observational data.

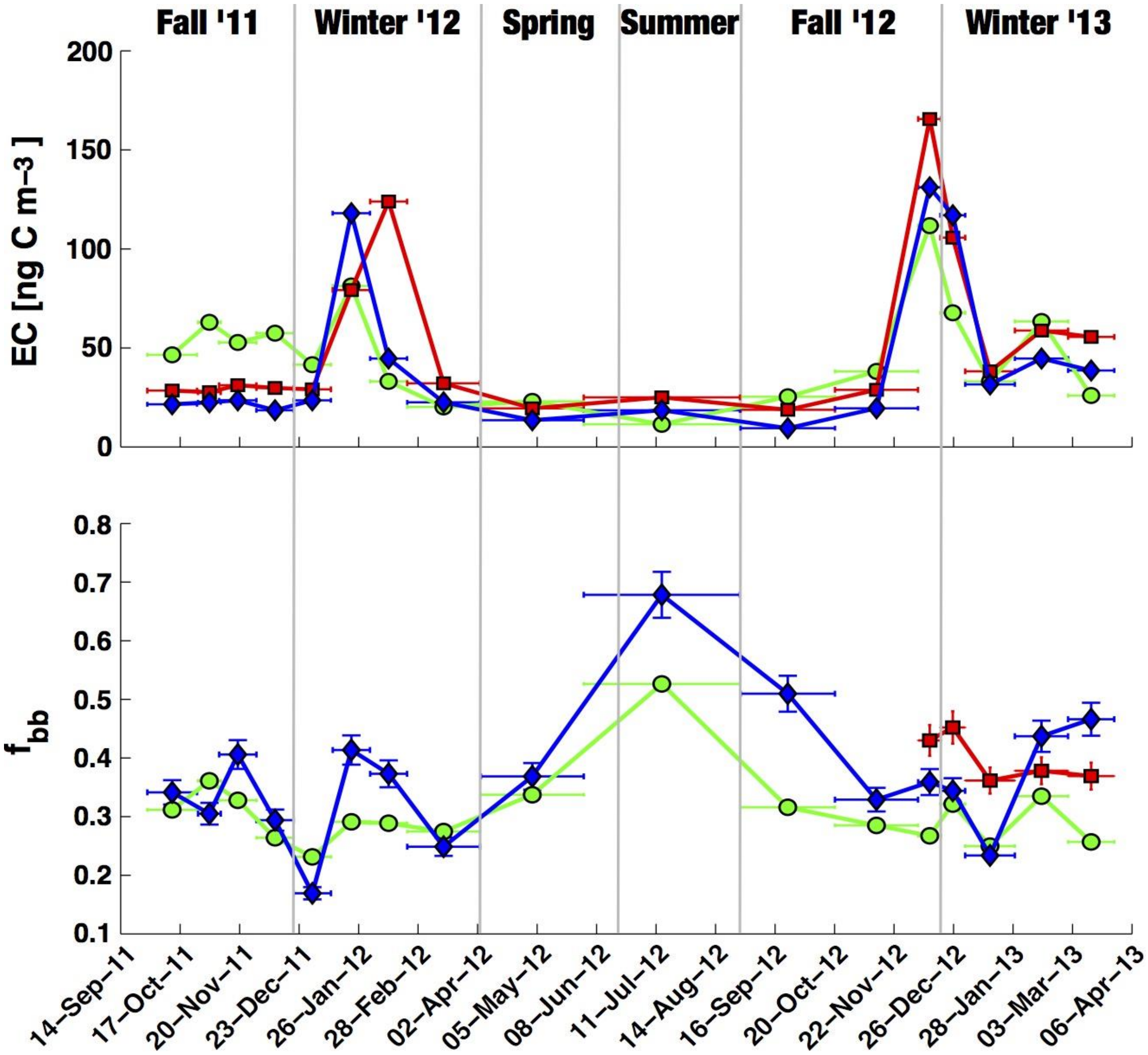

Supplementary Figure 3 TSP and $PM_{2.5}$ model comparison. a, Elemental carbon concentration for top-down measured $PM_{2.5}$ (blue line and diamond symbols) and TSP (red line and square symbols), and bottom-up BC concentrations simulated with FLEXPART driven with ECLIPSE emissions (green line and round symbols). Horizontal bars indicate duration of sampling. **b**, BC and EC based fraction of biomass burning for top-down measured $PM_{2.5}$ (f_{bb}, blue line and diamond symbols) and TSP (red line and square symbols), and bottom-up BC simulated with FLEXPART (green line and round symbols). Horizontal bars indicate duration of sampling. Vertical bars indicate standard

deviations of the observational data. The f_{bb} uncertainties shown for the TSP and $PM_{2.5}$ based f_{bb} are the results of the MCMC calculations.

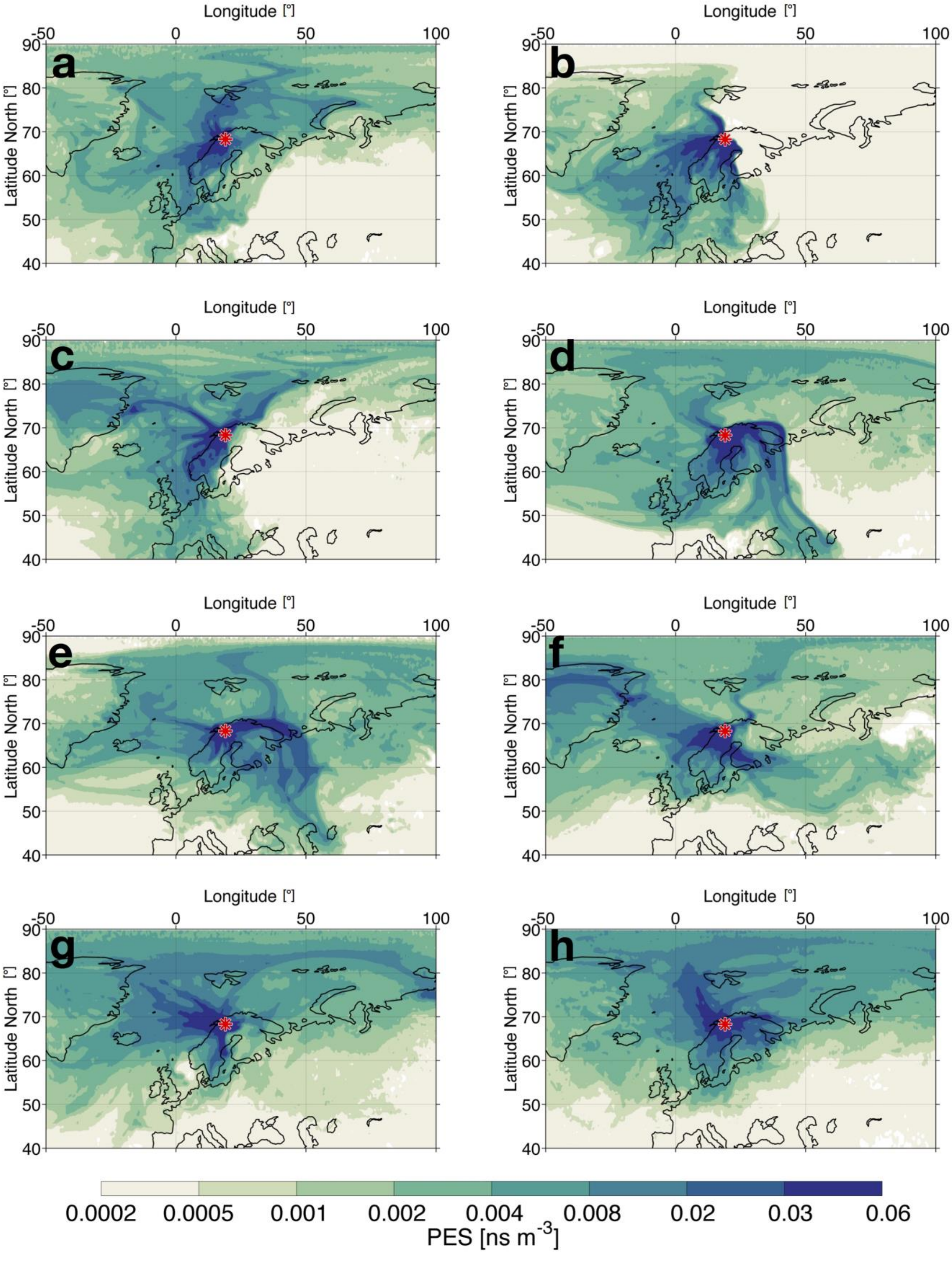
Longitude [°]
Latitude North [°]
-50
0
50
100
90
80
70
60
50
40
a
b
c
d
e
f
g
h
0.0002
0.0005
0.001
0.002
0.004
0.008
0.02
0.03
0.06
PES [ns m-3]

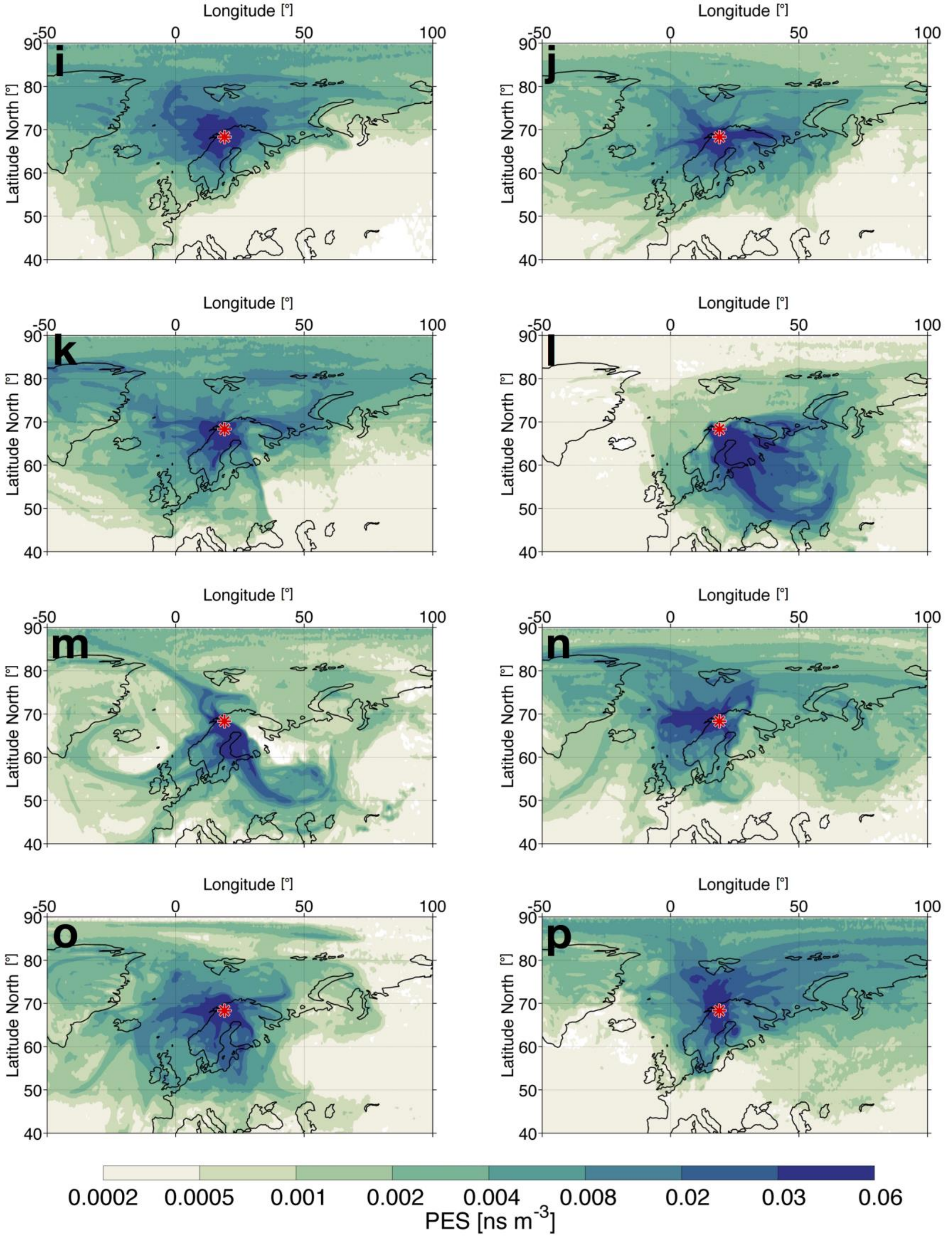

Supplementary Figure 4 FLEXPART potential footprint emission sensitivity (PES). PES for the black carbon aerosol tracer arriving at Stordalen mire, Abisko (red star). **a**, Isotope data point C1, 2011-09-29 to 2011-10-27. **b**, Isotope data point S1, 2011-10-27

to 2011-11-09. **c**, Isotope data point S2, 2011-11-09 to 2011-11-30. **d**, Isotope data point S3, 2011-11-30 to 2011-12-21. **e**, Isotope data point S4, 2011-12-21 to 2012-01-11. **f**, Isotope data point S6, 2012-02-02 to 2012-02-23. **g**, Isotope data point C2, 2012-02-23 to 2012-04-04. **h**, Isotope data point C3, 2012-04-04 to 2012-06-01. **i**, Isotope data point C4, 2012-06-01 to 2012-08-28. **j**, Isotope data point C5, 2012-08-28 to 2012-10-20. **k**, Isotope data point C6, 2012-10-20 to 2012-12-06. **l**, Isotope data point S7, 2012-12-06 to 2012-12-19. **m**, Isotope data point S8, 2012-12-19 to 2013-01-02. **n**, Isotope data point C7, 2013-01-02 to 2013-01-30. **o**, Isotope data point C8, 2013-01-30 to 2013-03-01. **p**, Isotope data point C9, 2013-03-01 to 2013-03-27.

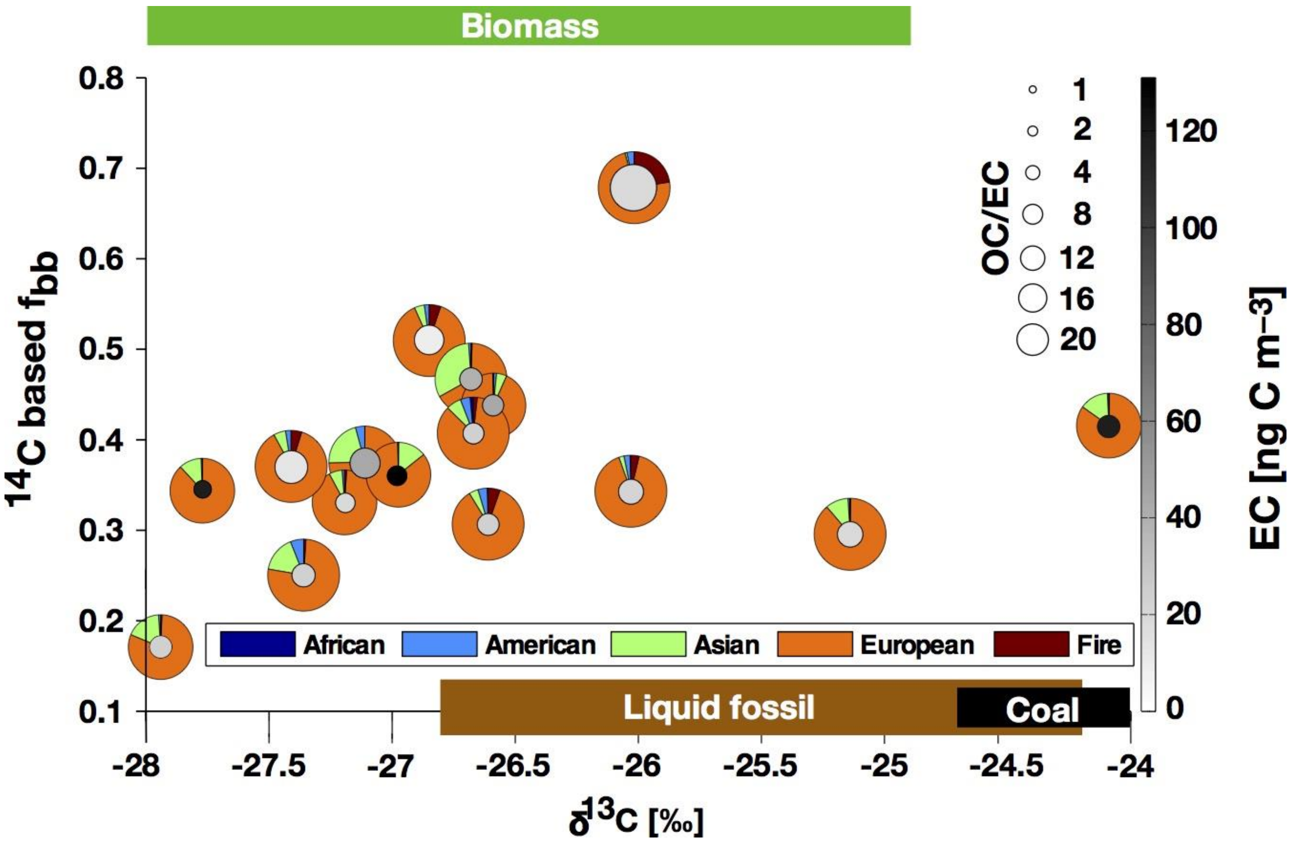

Supplementary Figure 5 Multi-Dimensional Source Apportionment. The expected $\delta^{13}C$ and $\Delta^{14}C$ endmember ranges for biomass burning emissions, liquid fuel combustion, and coal combustion are shown as green, brown, and black bars, respectively. The circles in grey scale show the respective EC concentrations, their area represent OC/EC ratios from 2 to 22 (see scale with black circles on upper right side). The coloured rings represent fractions of continental source regions for the anthropogenic contributions and emissions from open fires based on FLEXPART.

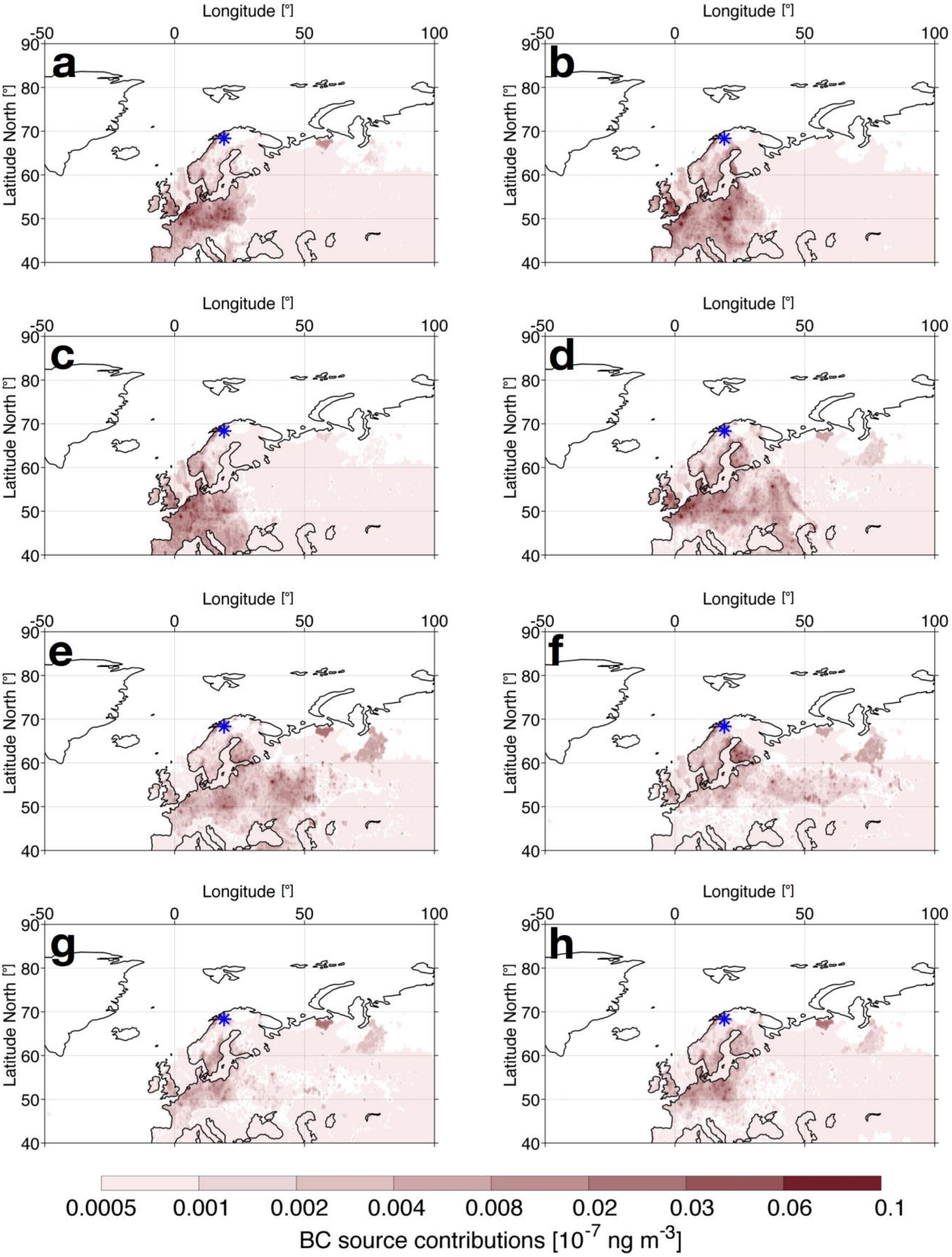
Longitude [°]
Latitude North [°]
a
b
c
d
e
f
g
h
-50
0
50
100
40
50
60
70
80
90
0.0005
0.001
0.002
0.004
0.008
0.02
0.03
0.06
0.1
BC source contributions [10^{-7} ng m^{-3}]

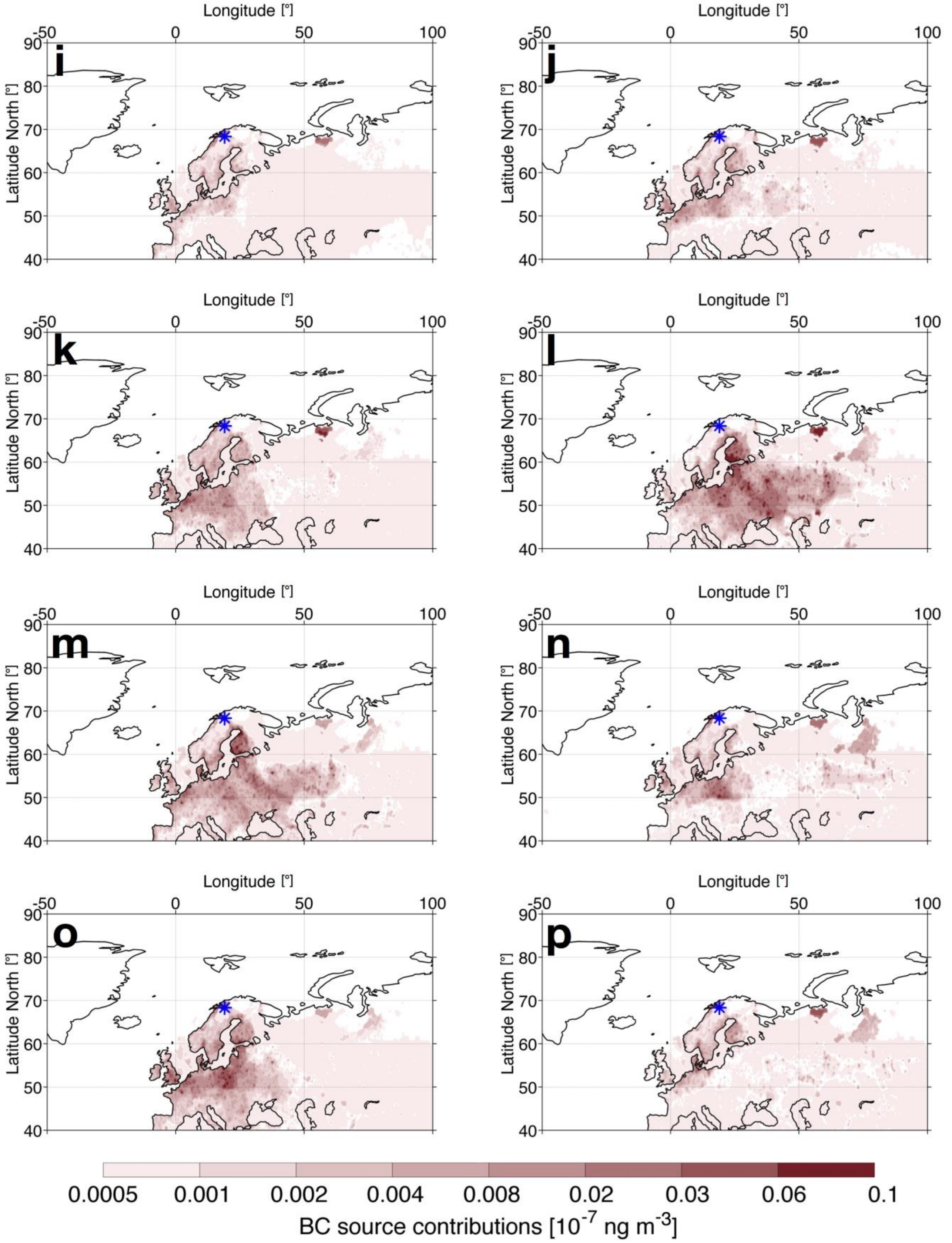

Supplementary Figure 6 FLEXPART geographical distribution. The anthropogenic BC source contribution to the simulated mixing ratio at Stordalen mire, Abisko (blue star).

a, Isotope data point C1, 2011-09-29 to 2011-10-27. **b**, Isotope data point S1, 2011-10-27 to 2011-11-09. **c**, Isotope data point S2, 2011-11-09 to 2011-11-30. **d**, Isotope data point S3, 2011-11-30 to 2011-12-21. **e**, Isotope data point S4, 2011-12-21 to 2012-01-11. **f**, Isotope data point S6, 2012-02-02 to 2012-02-23. **g**, Isotope data point C2, 2012-02-23 to 2012-04-04. **h**, Isotope data point C3, 2012-04-04 to 2012-06-01. **i**, Isotope data point C4, 2012-06-01 to 2012-08-28. **j**, Isotope data point C5, 2012-08-28 to 2012-10-20. **k**, Isotope data point C6, 2012-10-20 to 2012-12-06. **l**, Isotope data point S7, 2012-12-06 to 2012-12-19. **m**, Isotope data point S8, 2012-12-19 to 2013-01-02. **n**, Isotope data point C7, 2013-01-02 to 2013-01-30. **o**, Isotope data point C8, 2013-01-30 to 2013-03-01. **p**, Isotope data point C9, 2013-03-01 to 2013-03-27.

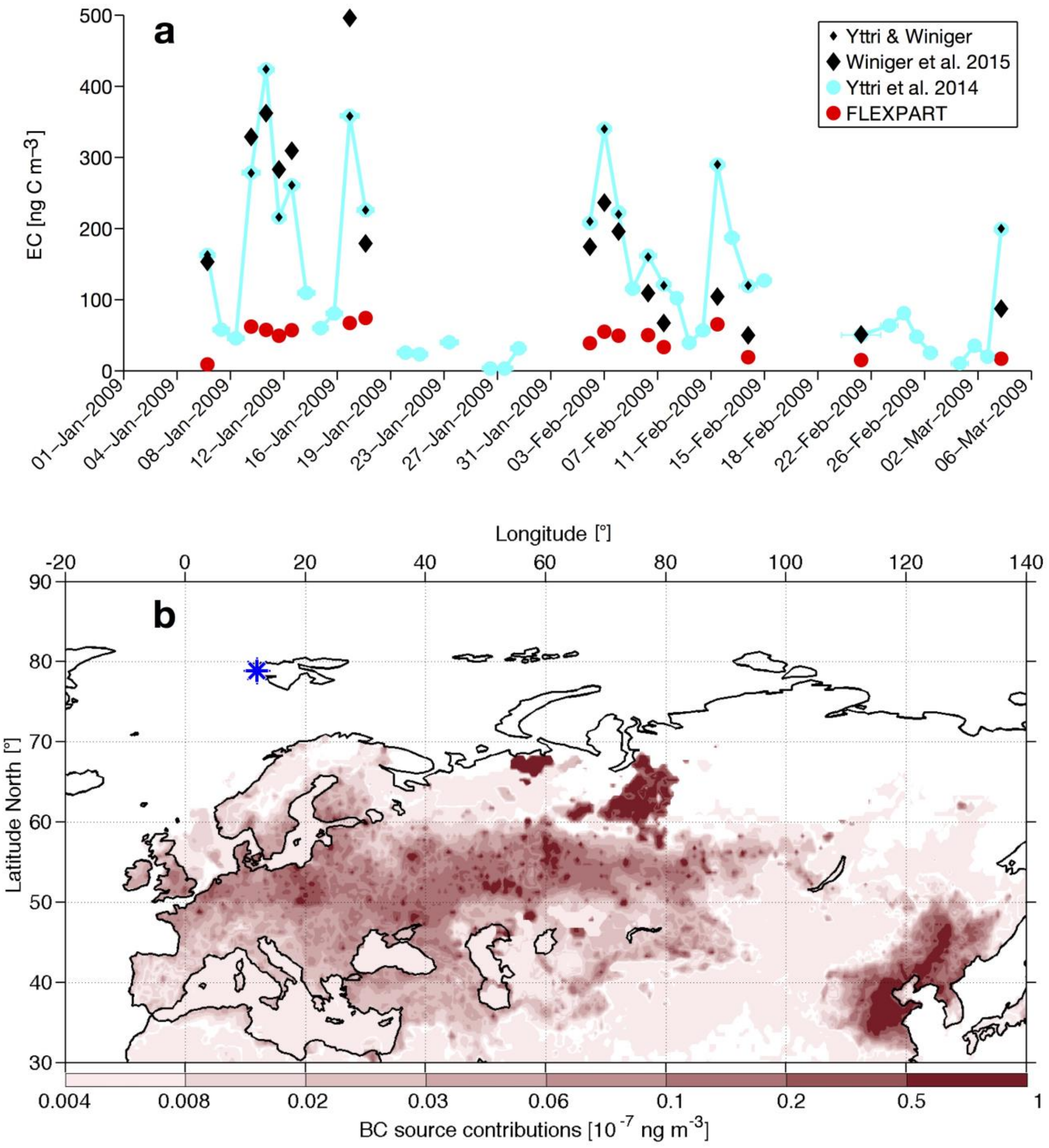

Supplementary Figure 7 Model - observation comparison for Zeppelin a, Elemental (black) carbon concentrations, measured at the Zeppelin Observatory. Original dataset by Yttri et al. [2014][1] (cyan circles), from which samples with highest EC concentration ('Yttri & Winiger') have been selected for radiocarbon measurements[2] (black diamonds). Model estimates by FLEXPART (red circles). **b**, Estimated anthropogenic BC

source contribution of all FLEXPART samples, measured at the Zeppelin Observatory (blue star).

Supplementary Tables

Supplementary Table 1 Seasonally averaged data for TSP and $PM_{2.5}$.

Season	Months	Start	Stop	$PM_{2.5}$				TSP			
		DD.MM.YY	DD.MM.YY	EC			OC/EC	EC			OC/EC
				[ng m^{-3}]				[ng m^{-3}]			
Fall '11	October-December	29.09.11	21.12.11	22	±	7.7	6	30	±	7.8	6
Winter '12	January-March	21.12.11	04.04.12	46	±	37	6	61	±	37	5
Spring	April-June	04.04.12	21.06.12	15	±	1.5	14	20	±	1.7	13
Summer	July-August	21.06.12	28.08.12	19	±	3.6	21	27	±	5.4	24
Fall '12	September-December	28.08.12	19.12.12	24	±	43	5	41	±	52	5
Winter '13	January March	19.12.12	27.03.13	50	±	30	4	59	±	24	3
Winter '12 & '13				48	±	33	5	60	±	30	4
Year 2012	January-December	21.12.11	19.12.12	27	±	34	8	40	±	39	8
All	Whole campaign	29.09.11	27.03.13	30	±	32	7	42	±	34	7

Supplementary Table 2 Stable carbon ($\delta^{13}C$) endmembers for different BC sources.

According to Andersson et al. (2015)[3].

BC source	$\delta^{13}C$ [‰]
Coal	−23.4±1.3
Liquid fossil fuels	−25.5±1.3
Biomass (C3)	−26.7±1.8
Gas flaring	−36 - −40

Supplementary Table 3 Isotope analysis of composites and single samples from ambient aerosol samples.

Sample	Start	Sampling	$PM_{2.5}$ EC			TSP EC		
ID		time	$\Delta^{14}C$	f_{bb}	$\delta^{13}C$	$\Delta^{14}C$	f_{bb}	$\delta^{13}C$
	DD.MM.YY	[d]	[‰]	[-]	[‰]	[‰]	[-]	[‰]
C 1	29.09.11	28.0	-580 ± 3	0.343 ± 0.012	-26.0 ± 0.2			
S 1	27.10.11	12.8	-625 ± 2	0.306 ± 0.012	-26.6 ± 0.2			
S 2	08.11.11	21.0	-501 ± 1	0.407 ± 0.011	-26.7 ± 0.2			
S 3	29.11.11	21.0	-638 ± 3	0.295 ± 0.012	-25.1 ± 0.2			
S 4	20.12.11	21.0	-790 ± 4	0.171 ± 0.014	-27.9 ± 0.2			
S 5	11.01.12	21.0	-492 ± 1	0.415 ± 0.011	-24.1 ± 0.2			
S 6	01.02.12	20.8	-542 ± 2	0.374 ± 0.012	-27.1 ± 0.2			
C 2	22.02.12	41.0	-693 ± 3	0.250 ± 0.013	-27.4 ± 0.2			
C 3	04.04.12	58.0	-547 ± 3	0.370 ± 0.012	-27.4 ± 0.2			
C 4	01.06.12	88.0	-169 ± 4	0.679 ± 0.013	-26.0 ± 0.2			
C 5	28.08.12	53.2	-375 ± 2	0.510 ± 0.012	-26.9 ± 0.2			
C 6	20.10.12	46.8	-595 ± 2	0.330 ± 0.012	-27.2 ± 0.2			
S 7	06.12.12	13.0	-559 ± 2	0.360 ± 0.012	-27.0 ± 0.2	-472 ± 2	0.431 ± 0.001	-27.0 ± 0.2
S 8	19.12.12	14.0	-577 ± 3	0.345 ± 0.012	-27.8 ± 0.2	-445 ± 2	0.453 ± 0.001	-26.9 ± 0.2
C 7	02.01.13	28.0	-712 ± 4	0.235 ± 0.013	-	-556 ± 3	0.363 ± 0.003	-26.7 ± 0.2
C 8	30.01.13	30.0	-463 ± 2	0.438 ± 0.012	-26.6 ± 0.2	-535 ± 1	0.379 ± 0.001	-26.5 ± 0.2
C 9	01.03.13	26.0	-428 ± 3	0.467 ± 0.012	-26.7 ± 0.2	-546 ± 1	0.370 ± 0.001	-27.3 ± 0.2

Supplementary Table 4 Quarterly top-down $PM_{2.5}$ observation compared to bottom-up estimates from FLEXPART-ECLIPSE.

Values with asterisk are means over the same study period (April-August).

Season	Months	Start	Stop	Observation		FLEXPART-ECLIPSE	
		DD.MM.YY	DD.MM.YY	$PM_{2.5}$ EC	f_{bb} based on $PM_{2.5}$ EC	EC	f_{bb}
				[ng m^{-3}]		[ng m^{-3}]	[-]
Fall '11	October-December	29.09.11	21.12.11	22 ± 7.7	0.345 ± 0.05	54	0.313
Winter '12	January-March	21.12.11	04.04.12	46 ± 37	0.349 ± 0.11	40	0.274
Spring	April-June	04.04.12	21.06.12	15 ± 1.5	0.579 ± 0.218*	17*	0.452*
Summer	July-August	21.06.12	28.08.12	19 ± 3.6	0.579 ± 0.218*	17*	0.452*
Fall '12	September-December	28.08.12	19.12.12	24 ± 43	0.388 ± 0.1	41	0.299
Winter '13	January March	19.12.12	27.03.13	50 ± 30	0.376 ± 0.11	46	0.289
Winter '12 & '13				48 ± 33	0.363 ± 0.11	43	0.278
Year 2012	January-December	21.12.11	19.12.12	27 ± 34	0.416 ± 0.15	31	0.353
All	Whole campaign	29.09.11	27.03.13	30 ± 32	0.397 ± 0.12	37	0.336

Supplementary Table 5 MCMC mean and standard deviation.

Sample ID	Start DD.MM.YY	Markov Chain Monte Carlo Biomass			Liquid fossil			Coal		
C 1	29.09.11	0.38	±	0.02	0.36	±	0.17	0.26	±	0.17
S 1	27.10.11	0.34	±	0.02	0.45	±	0.17	0.21	±	0.16
S 2	08.11.11	0.45	±	0.02	0.33	±	0.15	0.22	±	0.15
S 3	29.11.11	0.33	±	0.02	0.34	±	0.18	0.33	±	0.18
S 4	20.12.11	0.19	±	0.01	0.71	±	0.12	0.10	±	0.12
S 5	11.01.12	0.46	±	0.02	0.22	±	0.15	0.33	±	0.15
S 6	01.02.12	0.42	±	0.02	0.39	±	0.16	0.20	±	0.15
C 2	22.02.12	0.28	±	0.02	0.57	±	0.15	0.15	±	0.15
C 3	04.04.12	0.41	±	0.02	0.40	±	0.16	0.18	±	0.15
C 4	01.06.12	0.74	±	0.04	0.13	±	0.08	0.13	±	0.08
C 5	28.08.12	0.57	±	0.03	0.25	±	0.13	0.19	±	0.12
C 6	20.10.12	0.37	±	0.02	0.45	±	0.15	0.18	±	0.15
S 7	06.12.12	0.40	±	0.02	0.40	±	0.15	0.20	±	0.15
S 8	19.12.12	0.38	±	0.02	0.46	±	0.14	0.15	±	0.14
C 7	02.01.13		-			-			-	
C 8	30.01.13	0.49	±	0.03	0.30	±	0.15	0.22	±	0.15
C 9	01.03.13	0.52	±	0.03	0.28	±	0.14	0.21	±	0.13

Supplementary Table 6 Seasonal Results from the Dual-Carbon ($\Delta^{14}C$ and $\delta^{13}C$) Isotope-Based Bayesian Source Apportionment Calculations of BC. Values with asterisk are means over the same study period (April-August). The means for the whole campaign are missing one sample, where there was no $\delta^{13}C$ data (values marked with has tag).

		Start	Stop	MCMC								
Season	Months	DD.MM.YY	DD.MM.YY	Biomass			Liq. Fossil			Coal		
Fall '11	October-December	29.09.11	21.12.11	0.382	±	0.056	0.361	±	0.052	0.257	±	0.054
Winter '12	January-March	21.12.11	04.04.12	0.386	±	0.124	0.371	±	0.215	0.243	±	0.097
Spring	April-June	04.04.12	21.06.12	0.636	±	0.235*	0.219	±	0.194*	0.145	±	0.041*
Summer	July-August	21.06.12	28.08.12	0.636	±	0.235*	0.219	±	0.194*	0.145	±	0.041*
Fall '12	September-December	28.08.12	19.12.12	0.431	±	0.106	0.379	±	0.106	0.191	±	0.010
Winter '13	January March	19.12.12	27.03.13		-			-			-	
Year 2012	January-December	21.12.11	19.12.12	0.459	±	0.159	0.338	±	0.179	0.203	±	0.064
All	Whole campaign	29.09.11	27.03.13	0.449	±	0.126#	0.346	±	0.139#	0.205	±	0.063#

Supplementary Table 7 OC and EC analysis of ambient aerosol samples.

Start	Sampling time	$PM_{2.5}$							TSP						
		OC			EC			OC/EC	OC			EC			OC/EC
DD.MM.YY	[d]	[ng m^{-3}]			[ng m^{-3}]				[ng m^{-3}]			[ng m^{-3}]			
29.09.11	13.0	122	±	7.1	10	±	1.0	13	331	±	16	16	±	0.2	22
12.10.11	14.9	152	±	8.5	33	±	2.2	5	259	±	12	40	±	1.0	6
27.10.11	12.9	111	±	6.6	23	±	1.7	5	184	±	10	28	±	2.0	7
09.11.11	20.9	103	±	5.8	24	±	1.5	4	116	±	6.4	32	±	2.0	4
30.11.11	21.1	121	±	6.7	19	±	1.3	6	113	±	6.3	30	±	1.9	4
21.12.11	21.0	116	±	6.4	24	±	1.6	5	113	±	6.3	30	±	1.9	4
11.01.12	21.0	597	±	31	118	±	6.3	5	590	±	30	79	±	4.4	7
02.02.12	20.9	404	±	21	45	±	2.6	9	376	±	19	124	±	6.6	3
23.02.12	20.1	161	±	8.8	26	±	1.7	6	142	±	7.8	34	±	2.1	4
14.03.12	20.9	89	±	5.1	20	±	1.4	4	139	±	7.7	31	±	2.0	5
04.04.12	21.0	147	±	8.0	14	±	1.0	11	166	±	9.0	22	±	1.5	7
25.04.12	17.9	136	±	7.5	16	±	1.2	9	145	±	8.0	18	±	1.4	8
13.05.12	18.9	178	±	9.6	13	±	1.0	14	202	±	11	19	±	1.4	11
01.06.12	20.1	383	±	19	17	±	0.2	23	521	±	25	21	±	0.2	25
21.06.12	25.0	547	±	27	23	±	0.6	24	822	±	41	32	±	0.7	26
16.07.12	22.0	275	±	13	14	±	0.1	19	524	±	26	20	±	0.2	27
07.08.12	20.9	369	±	18	19	±	0.3	19	575	±	28	29	±	0.6	20
28.08.12	28.1	166	±	8.0	19	±	0.5	9	349	±	17	17	±	0.2	21
25.09.12	25.0	43	±	2.0	5.0	±	0.0	9	186	±	8.8	22	±	0.4	9
20.10.12	19.9	81	±	3.7	19	±	0.2	4	88	±	3.9	30	±	0.6	3

09.11.12	13.0	69	±	2.8	16	±	0.0	4	75	±	2.9	26	±	0.0	3
22.11.12	14.0	81	±	3.4	26	±	0.3	3	102	±	4.3	32	±	0.4	3
06.12.12	13.0	507	±	25	131	±	5.5	4	520	±	25	165	±	7.0	3
19.12.12	14.0	367	±	18	117	±	4.9	3	284	±	13	106	±	4.1	3
02.01.13	14.0	38	±	1.3	17	±	0.0	2	36	±	1.0	18	±	0.0	2
16.01.13	14.0	146	±	6.7	48	±	1.4	3	94	±	3.9	59	±	1.8	2
30.01.13	15.0	236	±	11	49	±	1.5	5	249	±	12	62	±	2.0	4
14.02.13	14.9	172	±	8.1	42	±	1.2	4	178	±	8.2	56	±	1.7	3
01.03.13	14.0	121	±	5.5	29	±	0.4	4	181	±	8.3	51	±	1.4	4
15.03.13	12.0	290	±	14	51	±	1.4	6	332	±	16	61	±	1.7	5

Supplementary Table 8 Emission partitioning of the ECLIPSE EI data. All available emissions were split according to their source type. Agricultural waste burning (on fields) is included in the Global Fire Emissions Database (GFED). This emission type was hence excluded from the used ECLIPSE emissions.

Biofuel	Fossil fuel	Fire (GFED)
Residential and commercial	Residential and commercial	Agricultural waste burning
Industry (combustion and processing)	Residential and commercial; non-fuel activity	
Power plants	Power plants, energy conversion, extraction	
	Industry (combustion and processing)	
	Industry (combustion and processing); non-fuel activity	
	Power plants	
	Power plants; non-fuel activity	
	Surface transportation	
	Waste	

Supplementary Table 9 FLEXPART-ECLIPSE data with observatory $PM_{2.5}$ EC as comparison.

Sample ID	Start	Sampling time	Observation		FLEXPART-ECLIPSE				
			$PM_{2.5}$ EC	f_{bb}	fossil EC	biofuel EC	fire EC	∑ EC	f_{bb}
	DD.MM.YY	[d]	[ng/m^3]	[-]	[ng/m^3]	[ng/m^3]	[ng/m^3]	[ng/m^3]	[-]
C 1	29.09.11	28.0	22 ± 17	0.343 ± 0.002	32	13	1.8	47	0.313
S 1	27.10.11	12.8	23 ± 1.7	0.306 ± 0.002	40	19	3.5	63	0.362
S 2	08.11.11	21.0	24 ± 1.5	0.407 ± 0.001	36	17	1.0	53	0.329
S 3	29.11.11	21.0	19 ± 1.3	0.295 ± 0.002	42	15	0.1	58	0.266
S 4	20.12.11	21.0	24 ± 1.6	0.171 ± 0.004	32	10	0.1	42	0.233
S 5	11.01.12	21.0	118 ± 6.3	0.415 ± 0.001	58	24	0.0	81	0.293
S 6	01.02.12	20.8	45 ± 2.6	0.374 ± 0.002	24	10	0.0	34	0.290
C 2	22.02.12	41.0	23 ± 4.5	0.250 ± 0.003	15	5.5	0.2	21	0.276
C 3	04.04.12	58.0	14 ± 1.4	0.370 ± 0.002	16	6.8	1.1	23	0.339
C 4	01.06.12	88.0	19 ± 3.8	0.679 ± 0.003	5.7	3.6	2.7	12	0.527
C 5	28.08.12	53.2	10 ± 10	0.510 ± 0.002	18	6.8	1.4	26	0.317
C 6	20.10.12	46.8	20 ± 5.0	0.330 ± 0.002	27	11	0.5	39	0.287
S 7	06.12.12	13.0	131 ± 5.5	0.360 ± 0.002	82	30	0.0	112	0.269
S 8	19.12.12	14.0	117 ± 4.9	0.345 ± 0.002	46	22	0.0	68	0.323
C 7	02.01.13	28.0	32 ± 22	0.235 ± 0.003	25	8.4	0.0	33	0.251
C 8	30.01.13	30.0	45 ± 4.6	0.438 ± 0.002	42	21	0.1	64	0.336
C 9	01.03.13	26.0	39 ± 15	0.467 ± 0.002	20	6.7	0.1	26	0.258

Supplementary Notes

Supplementary Note 1. Observation - model comparison for Zeppelin 2009

In previous work we investigated the $\Delta^{14}C$ signature of elemental carbon at the Zeppelin Observatory in Svalbard for the period January to March 2009[2]. For that period, no long-term filter collection was available, as in Abisko, but 24-hours samples. To obtain enough carbon material for radiocarbon AMS analysis these samples were therefore selected based on high EC concentrations only. On average, these samples displayed a rather high fraction biomass (52 ± 15%) for a winter period. The current FLEXPART-ECLIPSE-GFED model was applied to compare the Svalbard observations with modelling results (Supplementary Figure 7). Overall, the model suggested lower concentrations than observations and higher fossil contributions. We believe that a major reason for this offset is that the time points investigated by carbon-14 were influenced by relatively short and intense biomass burning events, which are difficult to being captured by the model[4]. The 'non-events' in the observation record were in similar range as the model predictions in terms of concentrations. We emphasize that high loading events are not likely to be large fires – which should be incorporated by GFED4 – since such are very unlikely during winter periods. Furthermore, we emphasize that the Svalbard study covers a limited time-period – in contrast to the year-round study in Abisko. Additionally, it is evident, that Zeppelin has very different sources, mainly Asian, compared to Abisko where European sources are dominant. This becomes important with regard to the emissions inventory, driving the FLEXPART model, which feature even higher uncertainties when it comes to Asia (specifically Russia[5]), as opposed to European emissions. Additionally, although the Zeppelin observatory is a ground based station, it is situated close to the top of Zeppelin mountain at 474 m a.s.l., which is an additional challenge to the model, since the observatory is not always inside the boundary layer, but inside the lower free troposhere[6].

Supplementary References

1. Yttri, K. E. *et al.* Quantifying black carbon from biomass burning by means of levoglucosan - A one-year time series at the Arctic observatory Zeppelin. *Atmos. Chem. Phys.* **14,** 6427–6442 (2014).
2. Winiger, P., Andersson, A., Yttri, K. E., Tunved, P. & Gustafsson, Ö. Isotope-Based Source Apportionment of EC Aerosol Particles during Winter High-Pollution Events at the Zeppelin Observatory, Svalbard. *Environ. Sci. Technol.* **49,** 11959–11966 (2015).
3. Andersson, A. *et al.* Regionally-varying combustion sources of the January 2013 severe haze events over eastern China. *Environ. Sci. Technol.* **49,** 2038–2043 (2015).
4. Schutgens, N. A. J. *et al.* Will a perfect model agree with perfect observations? The impact of spatial sampling. *Atmos. Chem. Phys. Discuss.* 1–32 (2016). doi:10.5194/acp-2015-973
5. Huang, K. *et al.* Russian anthropogenic black carbon: Emission reconstruction and Arctic black carbon simulation. *J. Geophys. Res. Atmos.* **120,** 11,306-11,333 (2015).
6. Tunved, P., Ström, J. & Krejci, R. Arctic aerosol life cycle: linking aerosol size distributions observed between 2000 and 2010 with air mass transport and precipitation at Zeppelin station, Ny-Ålesund, Svalbard. *Atmos. Chem. Phys.* **13,** 3643–3660 (2013).
